# Lignin as a Natural Carrier for the Efficient Delivery of Bioactive Compounds: From Waste to Health

**DOI:** 10.3390/molecules27113598

**Published:** 2022-06-03

**Authors:** Federico Verdini, Emanuela Calcio Gaudino, Erica Canova, Silvia Tabasso, Paria Jafari Behbahani, Giancarlo Cravotto

**Affiliations:** 1 Dipartimento di Scienza e Tecnologia del Farmaco, University of Turin, Via P. Giuria 9, 10125 Turin, Italy; federico.verdini@unito.it (F.V.); erica.canova@unito.it (E.C.); silvia.tabasso@unito.it (S.T.); paria.jafaribehbahan@edu.unito.it (P.J.B.); 2 Huvepharma Italia Srl, Via Roberto Lepetit 142, 12075 Garessio, Italy

**Keywords:** lignin, renewable material, extraction techniques, nanoparticles, drug carrier

## Abstract

Lignin is a fascinating aromatic biopolymer with high valorization potentiality. Besides its extensive value in the biorefinery context, as a renewable source of aromatics lignin is currently under evaluation for its huge potential in biomedical applications. Besides the specific antioxidant and antimicrobial activities of lignin, that depend on its source and isolation procedure, remarkable progress has been made, over the last five years, in the isolation, functionalization and modification of lignin and lignin-derived compounds to use as carriers for biologically active substances. The aim of this review is to summarize the current state of the art in the field of lignin-based carrier systems, highlighting the most important results. Furthermore, the possibilities and constraints related to the physico–chemical properties of the lignin source will be reviewed herein as well as the modifications and processing required to make lignin suitable for the loading and release of active compounds.

## 1. Introduction

**Figure 1:**
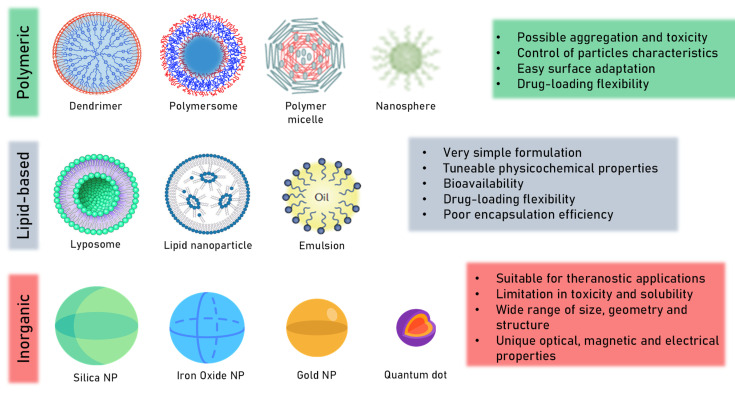
Classification of most common nanoparticles (NP) and their cargo, delivery and patient-response advantages and disadvantages.

**Figure 2:**
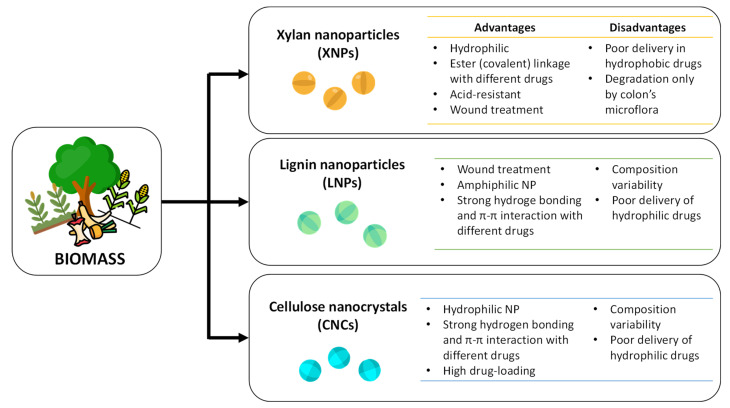
Pros and cons of the most common biomass-derived nanoparticles.

Lignocellulosic biomass is an earth-abundant resource (about 200 billion tons/year) that has much higher potential than is currently being exploited [1,2]. While it can be obtained from a variety of resources, it is essential to underline that biomass is a common industrial residue from food processing that has been exploited as a low-cost carbon source for the biorefinery. Moreover, the use of these residues as a feedstock does not compete with human food, making them an attractive alternative, considering current population growth [3]. However, in the last few years, there has been a change in the trend of lignocellulosic-biomass exploitation; from being a fuel source to other powerful applications, such as in advanced material synthesis, for the production of platform chemicals and even in some biomedical applications [4,5,6,7]. Nanoparticle-supported drug-delivery systems utilize several types of nanoparticles, both synthetic and bio-based, to store and deliver several drugs (Figure 1). Excellent results have been documented in the literature when using nanoparticle-supported delivery systems [8], as this approach provides better control of drug release and, consequently, of the side effects [9]. However, the toxicity and deformation of nanoparticles in the human body must be carefully considered. To date, many synthetic nanoparticles, such as silica nanoparticles [10], and metal oxides [11], have been successfully used as drug carriers, while those derived from natural sources have received less attention, although lignocellulosic-derived nanoparticles have recently gained increasing interest from the scientific community as drug-delivery systems, due to their biocompatibility, biodegradability and non-toxicity [12]. There are several kinds of lignocellulosic-derived nanoparticles that have been widely explored for drug-delivery systems, i.e., lignin nanoparticles (LNPs) [13,14], xylan nanoparticles (XNPs) [15], and cellulose nanocrystals (CNCs) (Figure 2) [16,17].

They have been developed from natural lignocellulosic biomass containing three main components, i.e., cellulose (35–50%), hemicellulose (20–35%) and lignin (5–30%) [1,12]. Of these three components, lignin aroused special attention as it is the most abundant and amorphous aromatic biopolymer. It plays a crucial role in plants, protecting them from microbial and chemical attacks, and providing mechanical support [18,19].

**Figure 3:**
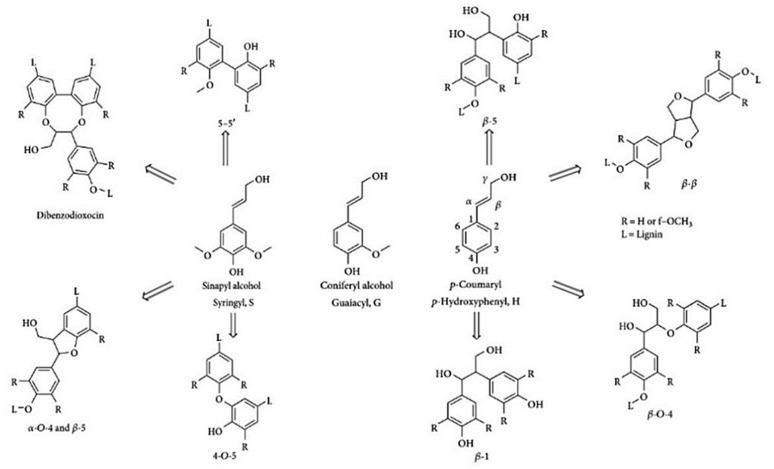
Lignin monolignols and common chemical bonds involved in the formation of lignin polymeric structure.

Typical lignin contents are 15–25% in grasses, 19–28% in hardwoods and 24–33% in softwoods. The molecular structure of lignin is highly dependent on the lignocellulosic species and, especially, on the extraction process from which it derives [19,20]. Besides its natural abundance, lignin is also present as a major by-product of the pulp and paper industry [21]. Different types of lignin contain different functional groups and possess different molecular weights and elemental compositions [22]. The functional groups in lignin include methoxy, carbonyl, carboxyl, and hydroxyl groups linked to aromatic or aliphatic moieties in various amounts and proportions, leading to different lignin compositions and structures. The structure of lignin is therefore extremely complicated and difficult to determine. Nevertheless, it is generally accepted that lignin is a three-dimensional and highly branched biomacromolecule that consists of three basic phenylpropane monomers, syringyl, guaiacyl and *p*-hydroxyphenyl [23], linked together by several bonds including several kinds of carbon–carbon and ether linkages (Figure 3) [24]. Nevertheless, there are many active functional groups in lignin, such as aliphatic and phenolic hydroxyl groups, carbonyl groups, methoxy groups and phenyl groups, that are important active sites for further chemical modifications via sulfonation, oxidation, graft copolymerization and hydroxymethylation reactions, etc. [25]. Weak β-O-4 chemical bonds are the most prevalent linkages in the lignin structure and are the main target of most degradation pretreatments, while other bonds, such as β-5, β-1, β-β′, 5–5 and 4-O-5, are more arduous and difficult to break (Figure 3).

Lignins from several degradation processes, such as milled wood lignin (MWL), acidic lignin, sulfite lignin, soda lignin, Kraft lignin, organosolv lignin, cellulolytic enzyme lignin (CEL), enzymatic mild acidolysis lignin (EMAL), and lignin from the thioacidolysis process, can be structurally examined. Nowadays, extraction and depolymerization with ionic liquids (ILs) are promising processes for the isolation of lignins [26], and the obtained modified lignin products can be utilized in several industrial fields [27], such as phenolic resins, adhesives, bioplastics and even in the fabrication of nanoparticles for use as drug carriers. The use of lignocellulosic biomass for nanoparticle synthesis, especially when it is derived from waste, is economically beneficial. However, a pretreatment step is mandatory due to the strong physical and chemical interactions between hemicellulose, cellulose, and lignin. Choosing a suitable sustainable biomass pretreatment method is therefore of crucial importance as it should not excessively increase costs [28,29]. This review presents the state of the art of lignin-derived nanoparticles for use in drug-delivery applications. In addition, the studies on the synthesis and characterization of these materials are critically discussed.

## 2. Lignin: From Extraction to Chemical Modification for Nanoparticles Synthesis

**Figure 4:**
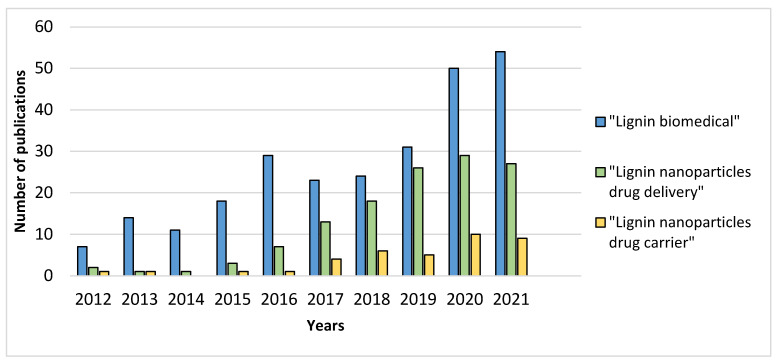
Number of publications per year on the use of lignin in the biomedical field (Web of Science^TM^).

The development and application of LNPs for drug delivery is increasing (Figure 4), but mainly depends on the source, chemical modification and physicochemical properties of the lignin used.

**Figure 5:**
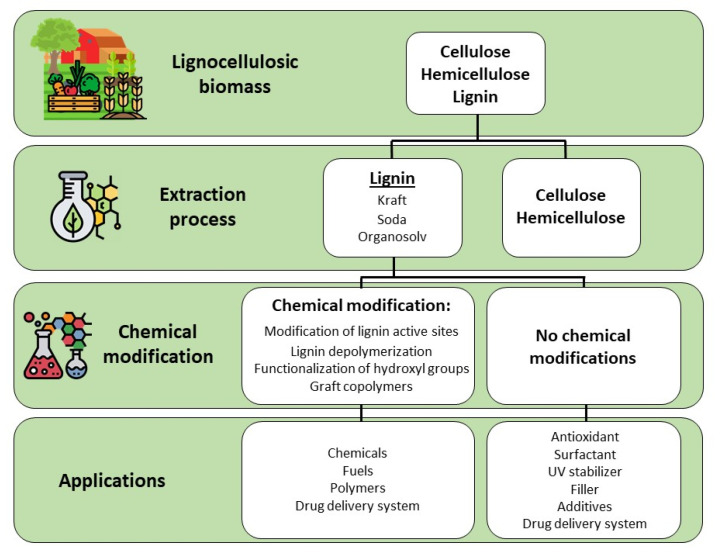
Schematic overview of lignin recovery, extraction, modification and applications.

**Figure 6:**
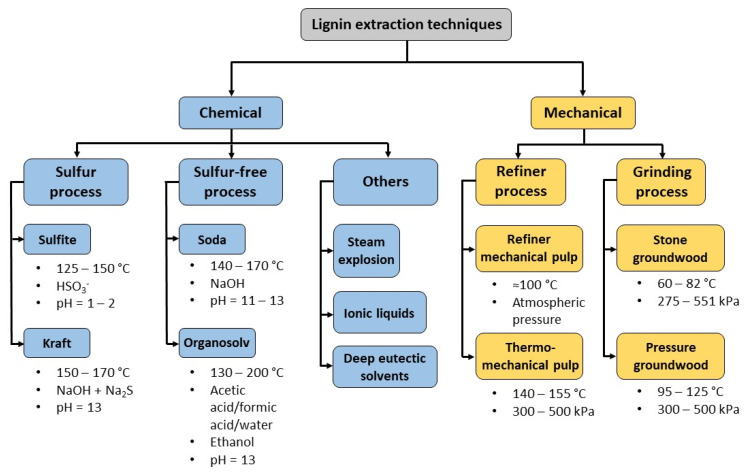
The most common lignin extraction techniques.

Romanì A. et al. [30], and Liao et al. [18], have described all the steps and methodologies for the successful extraction and modification of lignin from lignocellulosic-based wastes (Figure 5). Lignin is normally recovered using chemical extraction methods, such as solid-liquid extraction, which is frequently coupled with heat and/or agitation either using organic solvents, such as methanol, ethanol, butanol, ketones, ethers and polyols (organosolv lignin), or aqueous solutions of sodium hydroxide alone (alkali lignin) or in combination with sodium sulfide (Kraft lignin). Other traditional approaches include maceration, percolation, infusion, hot aqueous extraction, or decoction, Soxhlet extraction, hydro-distillation and mechanical processes such as grinding (Figure 6).

Furthermore, traditional methodologies are often time-consuming and employ toxic or dangerous organic solvents, meaning that it is important to use alternative methods for sustainable practice. Green methods include extractions using recyclable deep eutectic solvents and ionic liquids [31], electric fields, cavitation (acoustic and hydrodynamic cavitation) [28], subcritical and supercritical solvent conditions, enzymes, microwaves and green solvents [32], including eco-solvents such as glycerol and limonene and natural solvents (e.g., vegetable oils, bioethanol).

After the extraction procedures, lignin can be modified via several different chemical reactions. Four different approaches can be used to chemically modify lignin: (1) synthesis of new chemically active sites; (2) lignin depolymerization or fragmentation; (3) production of graft copolymers; and (4) chemical modification of the hydroxyl groups present in the lignin structure [23,33,34].

**Figure 7:**
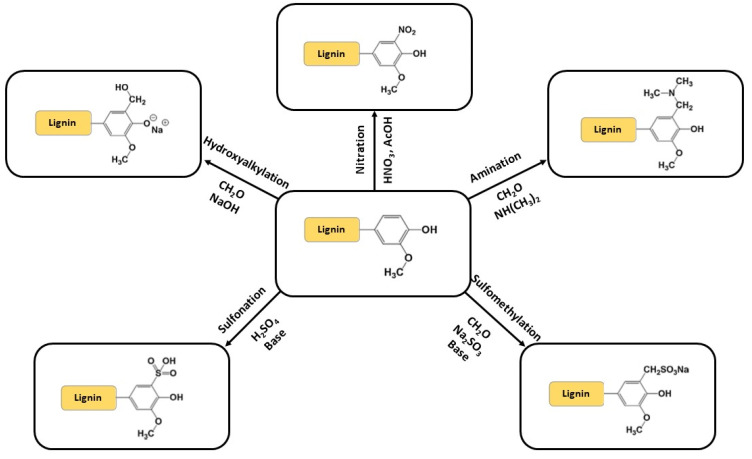
Synthesis of new chemically active sites by lignin chemical modification.

**Figure 8:**
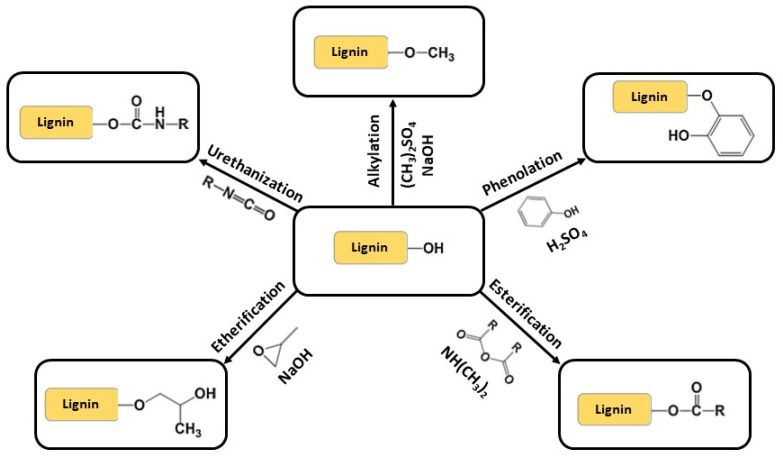
Lignin hydroxyl-group functionalization.

The modification of lignin active sites (1) consists of synthesizing new macromonomers that are more effective and reactive either by increasing the reactivity of the hydroxyl groups or by changing the nature of the chemically active sites, mainly via hydroxyalkylation, amination, nitration, sulfomethylation and sulfonation reactions (Figure 7), while the modification of the hydroxyl groups on the lignin structure (4) can lead to the formation of polyol derivatives of lignin via alkylation, esterification, etherification, phenolation and urethanization (Figure 8). Lignin graft copolymers (3) are commonly synthesized via ring-opening polymerization (reaction of lignin -OH groups with propylene oxide), radical polymerization (polymerization of vinylic monomers onto lignin), “grafting to” and “grafting on” techniques [35], and atom-transfer radical polymerization (formation of long polymer chains with well-defined structures and low dispersity) [36]. On the other hand, lignin can be depolymerized (2) via pyrolysis, oxidation to synthesize aldehydes (vanillin, syringaldehyde and their corresponding acids vanillic and syringic acid—using nitrobenzene, metallic oxides, oxygen and hydrogen peroxide as oxidizing agents) [37], hydrogenolysis and hydrolysis. 

### 2.1. Lignin Nanoparticles: Synthetic Methods

LNPs are renewable, biocompatible, nano-sized and structured aggregations of a lignin-derived biopolymer. Despite the lack of specific studies on LNPs biodegradability, it is assumed that LNPs are biodegradable as a resut of their natural biopolymeric composition [38] and the demonstrated ability of laccase and peroxidase enzymes to degrade LNPs polymeric structure [39,40]. Thanks to their hydrophobic core, composed of the lignin hydrophobic functional groups, and their hydrophilic shell, derived from the lignin hydrophilic functional groups, LNPs are a good carrier of hydrophobic molecules in high polarity environments. LNPs are a suitable choice for drug-delivery purposes due to their antimicrobial, anticancer, antioxidant anti-inflammatory properties, and their low cytotoxicity [41]. Lignin-based nanoparticles can be synthesized using several different techniques, such as acid precipitation, ultrasonication, solvent shifting and dialysis.

#### 2.1.1. Physical Method: Ultrasonication

Ultrasonication can be classified as a mechanical method for the production of LNPs. The phenomenon of US-induced cavitation (acoustic cavitation) consists of the formation, growth and subsequent collapse of cavitation bubbles, which generates high heat and local-pressure release. When ultrasound waves in the range of 20–150 kHz are applied to a liquid medium, the physical effects of cavitation, such as shockwaves and microjets, are predominant. On the other hand, ultrasound frequencies in the range of 150–2000 kHz mainly cause chemical effects due to the formation of hydroxyl radicals (HO·) in the local hotspots generated by cavitation [28]. The release of local pressure and the generation of oxidizing radicals can be exploited to disintegrate and depolymerize the macromolecular structure of lignin to obtain particles on a nanoscale level. For this purpose, Gilca et al. [42] sonicated an aqueous suspension of lignin (0.7%) with a US-horn for 60 min at a frequency of 20 kHz (600 W) to obtain a homogeneous and stable nanodispersion of LNPs with an average diameter of 0.03 µm. A similar treatment was performed by Gonzalez et al. [43] who synthesized LNPs from softwood kraft lignin. The water suspension of lignin (0.1% wt) was ultrasonicated at a frequency of 20 kHz, 130 W power and 95% oscillation amplitude with an ultrasonic tip for 2, 4 and 6 h. After each sonication experiment, the authors observed that the synthesized LNPs had an irregular shape, but they also found that a notable decrease in particle size occurred during treatment. In fact, after 6 h of treatment, they obtained lignin nanoparticles in the range of 10–50 nm starting from lignin agglomerates with dimensions in the 1–3 µm range. 

#### 2.1.2. Chemical Method: Acidic Precipitation

In the acidic precipitation method, nano-sized lignin particles are directly precipitated from a solution of dissolved lignin (e.g., black liquor) via the addition of an inorganic acid such as HCl, H_2_SO_4_ and HNO_3_. Frangville et al. [44] have obtained stable LNPs in a wide range of pH values by adding HCl to an ethylene glycol solution of lignin, and LNPs that are stable at low pH values by adding HNO_3_ to aqueous lignin solutions. Nano-sized lignin nanoparticles have also been synthesized in an ethylene glycol and water solution by Rahman et al. [45]. Moreover, the same authors have investigated the possibility of precipitating LNPs from castor oil via the dropwise addition of a 1 M HCl solution. A similar approach has been exploited by Pang et al. [46], to obtain LNPs from an alkaline aqueous lignin solution by decreasing its pH to 2.5. However, the solvent consumption of these techniques could limit their use at an industrial level. For this reason, Beisl et al. [47], have developed a method for LNP precipitation with the addition of H_2_SO_4_ directly from wheat straw organosolv extracts. 

Different approaches for LNP synthesis through the combined use of acid solutions and ultrasound have recently been developed. Agustin et al. [48], were the first to exploit different acidic water solutions (HCl, HNO_3_ and H_2_SO_4_) to recover precipitated lignin from birch-derived alkaline pulping liquor (APL). The lignin was subsequently dried, suspended in water and sonicated for 5 min at a frequency of 20 kHz (80% oscillation amplitude per 100 W). Sonication allowed the authors to collect monodispersed and stable nanoparticles. LNPs derived from natural resources and synthesized via a combination of acid precipitation and ultrasonication have also been obtained by Ingtipi et al. [49]. In detail, the authors initially dissolved solid, biomass-derived lignin using a 0.1 M solution of NaOH to separate the insoluble lignin by vacuum filtration, and then precipitated the soluble lignin using HCl. The so-obtained precipitated lignin was freeze-dried and sonicated for 3 h at a frequency of 20 kHz and a 40% oscillation amplitude (500 W) to recover nanoparticles in the 10–50 nm range. 

#### 2.1.3. Solvent Shifting

Solvent shifting is an environmentally friendly and controlled precipitation procedure that can be exploited to produce LNPs using low-cost materials. It relies on using a lignin solution and an excess amount of anti-solvent to reduce the solubility of lignin and, subsequently, produce nanoparticles. Behboudi et al. [50], have used ethanol as an antisolvent to recover lignin sulfonate nanoparticles from lignin sulfonate that had previously been emulsified into an aqueous solution of the Tween 80 surfactant. In particular, the authors synthesized lignin sulfonate nanoparticles with a size of 53 nm at low lignin sulfonate concentration (0.28 g/mL), moderate surfactant concentration (0.32 g/mL) and high anti-solvent quantity (92 mL of ethanol for 40 mL of the aqueous phase). A similar approach has been used, by Dai et al. [14], to synthesize magnetic and resveratrol-loaded lignin nanoparticles for use as a drug carrier. Alkali lignin (AL) was dissolved into different organic solvents, including methanol, ethanol and tetrahydrofuran, in the presence of resveratrol. Subsequently, spherical, well-dispersed loaded nanoparticles were obtained by adding a water solution of Fe_3_O_4_ as an antisolvent. The use of the AL/methanol solution allowed the authors to obtain perfect and uniformly spherical hollow nanoparticles that demonstrated enhanced in vitro resveratrol stability and release, better tumor reduction, drug accumulation and lower adverse effects than the free drug. Moreover, the solvent shifting method can also be coupled with ultrasound to improve the precipitation procedure. Alqahtani et al. [13], ultrasonicated the citrate buffer (anti-solvent) for a period of 10 min (700 W and 20 kHz) during the dropwise addition of previously dissolved lignin into a mixture of ethanol, water, and curcumin to produce curcumin-loaded lignin nanoparticles for wound-healing applications. The so-obtained nanoparticles exhibited strong in vitro antibacterial activity towards Gram-positive bacterial pathogens, while the in vivo results showed that wounded rats treated with curcumin-loaded LNPs exhibited better dermal-wound closure than the untreated control. Ultrasonication was also used by Wang et al. [51], during the development of a green and facile method for producing lignin nanoparticles. In detail, alkali lignin was initially modified in a microwave acetylation process without the use of any catalysts or solvents, aside from acetic anhydride, which acted as both a reaction reagent and dispersion solvent. Subsequently, the purified acetylated lignin was dissolved in THF solution at different concentrations (1.0, 2.0, 3.0 and 4.0 mg/mL) under different ultrasonication intensities (0, 100 and 200 W) to investigate the effect of sonication during the addition of water as an anti-solvent. The highest yield of regular lignin nanoparticles (82.3%) was obtained with a 4 mg/mL initial concentration of acetylated lignin of under an ultrasonic intensity of 200 W. The combination of the solvent-exchange method and ultrasound was exploited by Cao et al. [52], to produce LNPs that could tune the mechanical properties of poly(vinyl alcohol). Kraft lignin was dissolved in ethanol by ultrasonic dispersion and recovered in the form of spherical nanoparticles thanks to the dropwise addition of distilled water. Subsequently, LNPs were recovered thanks to a dialysis procedure and added to the poly(vinyl alcohol) matrix, improving its toughness and strength to 6.33% and 289.9%, respectively. 

Ultrasonication and solvent-exchange techniques have been performed separately on the same matrix and their efficiencies in the production of LNPs from alkali lignin were compared by Camargos et al. [53]. In detail, the authors discovered that the use of an antisolvent alone led to the synthesis of nanosized spherical and regular LNPs in contrast to what they obtained with the ultrasonication alone, confirming that the combination of the two methods represents the best solution to enhance the LNPs production up to now. Despite the widespread use of combined ultrasound and solvent exchange as a precipitation procedure, some authors [54,55], have investigated the possibility of combining dialysis techniques and the solvent exchange method. 

#### 2.1.4. Deep Eutectic Solvents

Deep eutectic solvents (DESs) and bio-based DESs (natural deep eutectic solvents, NaDES) consist of a mixture of a hydrogen bond acceptor (HBA), often a quaternary ammonium salt, and a hydrogen bond donor (HBD), such as alcohols, acids, amines or carbohydrates. These special solvents are used to improve the pretreatment of lignocellulosic biomasses, often in combination with cavitational treatments, to facilitate the isolation and recovery of the cellulose fraction, with the lignin previously being dissolved into DESs [28]. Xie et al. [56], have investigated process intensification strategies for the pretreatment of Radiata Pine with a recyclable green DES system, which is composed of benzyltrimethylammonium chloride/formic acid (BTMAC/FA). The lignin that was dissolved into the DES was recovered and characterized using imaging analysis software that allowed the authors to measure the height and diameter of the nanoparticles. They obtained oblate spherical or disk-shaped nanoparticles with a diameter of 1 μm and corresponding heights of approximately 10 nm. Recyclable DESs have also been used by Luo et. al. [57], who have developed a simple and sustainable protocol to produce LNPs by dissolving industrial lignin into different deep eutectic solvents and then inducing nanoprecipitation by the addition of H_2_SO_4_ or NaOH to test the effect of pH on LNP morphology. In detail, the authors tested various DESs, including an acidic NaDES (choline chloride:lactic acid 1:9), a polyol-based DES (choline chloride:ethylene glycol 1:2), and an alkaline DES (choline chloride:ethanolamine 1:6) for their ability to dissolve lignin. They discovered that increasing the pH and reducing the pre-concentration of lignin decreases the nanoparticle diameter, preserving their chemical structure and molecular weight. The same authors also tested the combined use of DESs, ultrasonication and solvent shifting to prepare LNPs from alkali lignin [58]. Choline chloride (ChCl) and ethanolamine (ETA) were used to synthesize alkaline DES at a molar ratio of 1:6, while choline chloride (ChCl) and lactic acid (LA) were used to prepare acidic NaDES at a molar ratio of 1:9. The lignin solutions were sonicated for 20 min at 240 W and the so-obtained LNPs were recovered by dialysis. The LNPs that were produced in the alkaline DES solvent were smaller in size than those recovered from the acid DES system and showed good dispersibility. Choline chloride (ChCl) and lactic acid (LA) NaDES have also been used to dissolve and recover lignin directly from the starting residual lignocellulosic biomass by Luo et al. [59]. In this case, wheat straw was initially suspended in DES and heated under stirring for several hours. The treated homogenate was then filtered to remove the residual solid and to collect the DES soluble lignin fraction, which allowed the authors to extract high purity lignin and recover the well-dispersed LNPs, which displayed a narrow size distribution that peaked at 70–90 nm. 

## 3. Examples of Lignin-Based Nanoparticles for Drug Delivery

Interest in the development and use of polymeric, inorganic and lipid-based nanoparticles (Figure 1)—also defined as “solid colloidal particles”—in medicine and drug delivery is rapidly increasing. This is especially true for cancer therapy as free therapeutics have several limitations such as biodistribution, biological barriers, molecular transport, and specific targeting. A reduction in toxicity, while maintaining the therapeutic effect, more specific drug delivery and targeting, and greater safety and biocompatibility, are therefore important objectives for nano-bio-technology research in the drug delivery field. Promising in vitro and small animal model experiments have revealed the potential of NPs to enhance the solubility and stability of encapsulated drugs, promote membrane transport and extend circulation times [60,61]. However, it is necessary to consider the possible adverse effects of residual NP materials post drug delivery, and, for that reason, natural and biodegradable nanoparticles, such as lignin, dextran, alginates and starch nanoparticles may be sustainable candidates for the development of nano drug-carrier systems. Biodegradable NPs are currently used as promising drug-carrier devices, as carriers of DNA in gene therapy and to deliver peptides, proteins and genes via oral administration [62]. Of the natural and biodegradable drug carriers available, lignin nanoparticles (LNPs) appear to be the most promising green tool for drug delivery as a result of the low-cost of the raw material, the simple and controllable conversion into uniform NPs, despite lignin heterogeneous chemical structures [63], and the enhanced antioxidant activity, which is due to the higher surface-area-to-volume ratio, when converted into NPs [64]. 

Nevertheless, the use of lignin as a drug nanocarrier is still a huge challenge. In fact, it is known that the antioxidant and antibacterial activities, biocompatibility (or cytotoxicity) and biodegradability inside the human body of LNPs depend on several factors, including biomass source, extraction methods, post-treatment reactions, type of lignin and microbial species [65,66,67,68,69,70]. For example, Alzagameem et al. [71], have evaluated different kinds of lignin and found that the organosolv lignin of softwood possessed higher antimicrobial activity than both Kraft lignin from softwood and organosolv lignin from grass, while Kaur et al. [72], have established that unmodified sugarcane bagasse lignin showed more effective antioxidant activity than acetylated and epoxidized chemically modified lignin. Several authors have performed in vitro experiments on specific biomass-derived LNPs to investigate their cytotoxicity [64,73,74,75]. However, the reported studies were conducted separately for each different type of lignin, and, for this reason, more studies would be needed to obtain the fundamental knowledge of lignin’s impact on the human body. The reviewed applications of lignin for biomedical use as a drug carrier are summarized in Table 1.

Resveratrol (RSV) is a natural compound that has been intensively investigated in recent years for its potential use in the pharmaceutical field [76]. However, the strong hydrophobicity of RSV limits its applications, making its encapsulation into nano-drug carriers a possible solution. Dai et al. [14], have synthesized RSV-loaded magnetic alkali lignin nanoparticles (AL/RSV/Fe_3_O_4_ NPs) to investigate their possible application as a stable nanodrug carrier for anticancer treatment. Firstly, the release of RSV from the AL NPs was analyzed using a dialysis method, and the encapsulation efficiency (DLE) and drug-loading capacity (DLC) were calculated according to the following equations:

**DLE** (%) = (weight of RSV in nanoparticles/weight of RSV added initially) × 100% (1)

**DLC** (wt%) = (weight of RSV in nanoparticles/weight of RSV-loaded nanoparticles) × 100% (2)

The results of RSV payload and the in vitro release profile showed relatively high encapsulation efficiency (DLE > 90%), drug loading capacity (DLC > 20%) and slow-release kinetics in AL NPs, probably due to the strong interaction between the RSV molecules and the carrier material. Moreover, the RSV-loaded LNPs have shown good anticancer effects, better tumor reduction and less severe adverse effects than free drugs in animal and cytological tests. This green and renewable material with its easy large-scale production and simple preparation is a candidate with huge potential for many poorly water-soluble drugs. Another interesting nanodrug carrier for anticancer drugs has been synthesized by Chai et al. [77], using a simple microfluidic system. Lignin/chitosan nanoparticles (Lig/Chi NPs) with an average particle size of about 180 nm were prepared using a solvent shifting process by dissolving the lignin extracted from corncob powder (formic acid pretreatment) in tetrahydrofuran and using a chitosan water solution as an antisolvent in a valve-assisted mixer supplied with a medium pressure flow pump. The carboxyl groups of lignin and the amino groups of chitosan facilitated the electrostatic co-assembly of the two partners. Two hydrophobic anticancer drugs, curcumin (CCM) and docetaxel (DTX), were co-assembled in the LNPs, and in-vitro drug-release determination (dialysis) and toxicity analyses (CCK-8 assay on HeLa cells) were subsequently performed. Both the DTX@Lig/Chi NPs and CCM@Lig/Chi NPs displayed a killing effect on the HeLa cells, and the drug-release amounts in acidic solutions that simulated the tumor microenvironment were 51% and 50%, respectively.

Siddiqui, et al. [78], have developed a lignin self-assembling technique to optimize the particle size of Blank Lignin Nanoparticles via simultaneous solvent exchange and flash pH change (BLNPs). To prepare the LNPs, Kraft lignin was dissolved in DMSO and the soluble fraction was then dialyzed in two steps; firstly, in dilute aqueous HCl (pH 5.3) to assist lignin self-assembly into nanoparticles and, secondly, in deionized water. Spherically shaped LNPs with controllable size (~152 nm) and low polydispersity were obtained. Irinotecan (a water insoluble anticancer base) was selected as a model drug and was encapsulated into the LNPs during self-assembly via dissolution in DMSO together with alkali lignin. Hemocompatibility, cytotoxicity and genotoxicity studies were performed on *Drosophila Melanogaster*, used as a model organism, to investigate BLNP efficiency as a drug carrier. In order to assess the anti-proliferative action of Irinotecan-loaded LNPs, in vitro studies were performed on a human breast adenocarcinoma cell line, a human alveolar epithelial adenocarcinoma cell line and a human embryonic kidney cell line. In vitro cytotoxicity studies showed that BLNPs were substantially toxic (74.38 ± 4.74%) to human breast adenocarcinoma (MCF-7), marginally toxic (38.8 ± 4.70%) to human alveolar epithelial adenocarcinoma (A-549) and insignificantly toxic (15.89 ± 2.84%) to human embryonic kidney (HEK-293) cells.

Zhou et al. [79], have obtained Acetylated Lignin (ACAL) and Benzoylated Lignin (BZAL) from pine alkali lignin through chemical modification. Alkali lignin was first dissolved in N, N-dimethylformamide (DMF) and, subsequently, triethylamine (catalyst), and acylation reagents (acetyl chloride or benzoyl chloride) were added. The reaction mixture was heated at 50 °C for 3 h and excess deionized water was added at the end of the reaction. The new reaction system was further heated at 90 °C for an hour to precipitate and recover the acylated alkali lignin. ACAL and BZAL were used to encapsulate avermectin (AVM), which is a pesticide that is generally used in animal husbandry. Thanks to the acylation-induced hydrophobicity of AL, the compatibility between AL and AVM was increased. The controlled release behavior of AVM-loaded nanospheres was studied and AVM@BZAL was observed to be better than AVM@ACAL in terms of its release properties. In addition, since AVM can be degraded by UV irradiation, the LNPs can protect the pesticide from photo-oxidative decomposition. For this purpose, photodegradation studies were performed, and it was found that both AVM@ACAL and AVM@BZAL possess good anti-photolysis properties. 

Avermectin was also encapsulated into a lignin-based nanocarrier by Peng R. et al., [80]. Specifically, the authors prepared a sodium lignosulfonate (SL)-cetrimonium bromide (CTAB) nanocarrier (AL/CTAB) in a self-assembling process, in which a SL aqueous solution and the CTAB ethanolic solutions were mixed. Once the SL/CTAB system was recovered, the avermectin nano-formulation was prepared by initially dissolving both SL/CTAB and avermectin in ethanol under ultrasonication and, finally, adding water under stirring. The nano-formulation showed controlled-release behavior, and the cumulative release amounts ranged from 56.27% to 87.33% in 62 h. Furthermore, after irradiation with UV for 50 h, the AVM retention rates in the nano-formulation ranged from 46.67% to 63.41%, which is 2.18–2.96 times higher than the commercial avermectin Emulsifiable Concentrate (EC).

**Figure 9:**
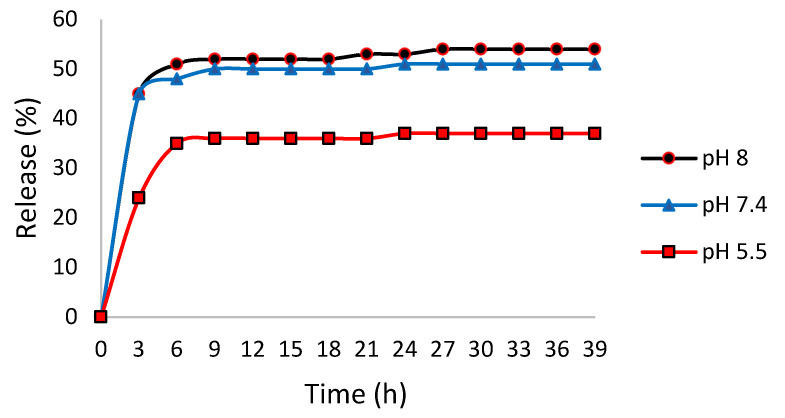
pH-controlled drug delivery of ASKL-chol.

Zhaleh P. et al. [81], have created a new biomaterial for the delivery of hydrophobic molecules from two fractions of Kraft lignin (low and high molecular weight) reacted with cholesteryl chloroformate (CholCl). In order to produce the low and high molecular weight fractions, the starting alkali lignin was mixed with pure acetone, and the acetone-soluble fraction (ASKL, low molecular weight) was separated from the acetone-insoluble fraction (AIKL, high molecular weight). Subsequently, cholesteryl chloroformate in dry dimethylformamide (DMF) was added to the dissolved lignin in DMF (both lignin fractions), to perform its encapsulation, after which pyridine was added and stirred for 24 h at room temperature. At the end of the reaction, DMF was removed by centrifugation, and the pyridine was removed through the addition of 50 mL HCl (5 mol/L), 5 wt% NaHCO_3_, de-ionized water and 30 mL brine, and both the AIKL-chol and ASKL-chol nanodrug carriers were recovered. Dynamic Light Scattering (DLS) studies showed an average particle size of 500 nm for AIKL-chol nanodrug carrier, and of 320 nm for the low molecular weight-AL analogue. ASKL-chol particles were chosen for folic-acid (hydrophobic drug) release experiments due to their smaller size and narrower distribution. With a loading efficiency of ∼67%, the ASKL-chol drug carrier displayed preliminary evidence of pH-controlled drug delivery (Figure 9). 

Patricia F. et al. [82] investigated three types of lignin nanoparticles (LNPs) as biodegradable nanodrug carriers: pure lignin nanoparticles (pLNPs); iron (III)-complexed lignin nanoparticles (Fe-LNPs); and Fe_3_O_4_-infused lignin nanoparticles (Fe_3_O_4_-LNPs). pLNPs were obtained via a dialysis process, while the Fe-LNPs were synthesized by adding a Fe(OiPr)_3_ (iron (III) isopropoxide) solution in THF to a solution of lignin in THF, to perform a condensation reaction. For the preparation of Fe_3_O_4_-LNPs, however, a 50:50 *w*/*w* mixture of lignin solution together with oleic acid coated Fe_3_O_4_ NPs in THF was prepared and then dialyzed against deionized water. All the synthesized drug-delivery systems were round-shaped, showed a narrow size distribution, reduced polydispersity and good stability at pH 7.4. In addition, in vitro cytotoxicity studies on a human umbilical vein cell line (EA.hy926), human mammary carcinoma cell lines (MDA-MB-231 and MCF-7), a human prostate cancer cell line (PC3-MM2), a human colorectal adenocarcinoma cell line (Caco-2) and a human myeloid cell line (KG1) demonstrated that LNPs had low cytotoxicity and hemolytic rates in all the tested cell lines. In order to investigate drug loading into the pLNPs, two slightly water-soluble cytotoxic agents, benzazulene (BZL) and sorafenib (SFN), and a water-soluble drug, capecitabine (CAP), were used as model drugs. The two poorly water-soluble drugs were efficiently loaded into pLNPs and their release profiles at pH 5.5 and 7.4 were improved. Moreover, the nanodrug carrier BZL-pLNPs showed an improved antiproliferation effect, compared to BZL alone, in several cells.

Another promising therapeutic agent has been prepared by M.B. Marulasiddershwara, et al., through green synthesis [83]. Lignin Capped Silver Nanoparticles (LCSN) were synthesized by suspending alkali lignin in water and, subsequently, mixing the suspended lignin with AgNO_3_ at 100 °C for 4–5 h under reflux. The dark grey powder of LCSN was obtained after the removal of the solvent. The therapeutic agent showed antibacterial and antifungal activity against human pathogens *S. aureus*, *E. coli* and *A. niger* and the percentage of zones of inhibition were found to be 10%, 12% and 80%, respectively. Additionally, LCSN was also revealed to have antioxidant behavior and the percentage activity against positive control vitamin C was found to be 70%. 

Zikeli F. et al. [84], have developed an interesting nanodrug carrier based on lignin nanoparticles (LNPs) loaded with essential oils (EO) derived from cinnamon bark (*Cinnamomum zeylanicum* Blume), common thyme (*Thymus vulgaris* L.) and wild thyme (*Thymus serpyllum* L.). These essential oils were chosen due to the presence of effective biocides against wood-decaying bacteria, such as thymol, cinnamaldehyde, eugenol and carvacrol. Nonetheless, EOs in wood-preserve formulations show instable activity and high sensitivity to oxidation and photodegradation, making the introduction of LNPs crucial to protect the EOs from degradation. The authors isolated acidolysis lignin from beech wood sawdust and prepared the loaded nanodrug carrier using a fast solvent-exchange method. Specifically, lignin was dissolved in DMSO, and the essential oils were subsequently and separately added to the lignin solution. EO-LNPs were recovered by filling dialysis bags with the so-obtained solutions against 4 L of distilled water for only 1 h to avoid the release of entrapped EOs. Py-GCMS and FTIR spectroscopy revealed the strong molecular interactions between the essential oil compounds and LNPs. Essential-oil loading, drug-loading efficiency (DLE) and drug-loading capacity (DLC) were also investigated, and the results are summarized in Table 2.

The in vitro release experiments for the EOs from common thyme and wild thyme entrapped in LNPs showed significantly delayed EO leaching from the lignin carrier material due to the strong molecular interaction between the two compounds. The essential oil from cinnamon bark was less compatible for entrapment in LNPs. The results obtained by Zikeli F. et al. will serve as a solid base for the development of a promising new green biocide delivery system. 

Wang et al. [85], have established a new effective preparation process for the synthesis of homogeneous and size-controllable pH-responsive LNPs and their application in oral drug delivery. Industrial alkali lignin was dissolved in a mixture of acetone and water (4:1) and LNPs were obtained via vacuum evaporation, with the effect of bath temperature, cooling temperature, lignin concentration and starting volume being investigated using Box–Behnken experimental design (BBD). Homogenous LNPs with a targeted size (within 100~400 nm) were recovered under the preparation conditions suggested by the established models. In order to investigate the drug-carrier properties of the so-obtained LNPs, ibuprofen (IBU) was encapsulated during the preparation of the nanodrug delivery system, starting with a mixture of ibuprofen: lignin 1:1. The matrix entrapment efficiency of LN was 47% and the IBU-LNP showed excellent pH-responsive release; only 18% was released at pH 1.2, while over 90% was released at pH 7.5 for 2−6 h.

Zhao J. et al. [86], have developed self-assembling nanoparticles that consist of an aminated lignin-histidine conjugate and the anti-tumor drug 10-hydroxycamptothecin (AmL-HIS/HCPT NPs) for efficient drug delivery. In order to achieve triggered drug release in the acidic tumor-cell microenvironment, the NPs are based on histidine, a pH-responsive molecule. Aminated lignin (AmL) was synthesized using the Mannich reaction, with the commercial Kraft lignin firstly being dissolved in water and, secondly, with diethylenetriamine (DETA) and formaldehyde being added in accordance with the reaction. The conjugation between AmL and histidine (HIS) was achieved by dissolving AmL in water and adding 1-(3-dimethylaminopropyl)-3-ethylcarbodiimide hydrochloride (EDC·HCl) and *N*-hydroxysuccinimide (NHS). The reaction mixture was stirred for 3 h at room temperature, subsequently dialyzed and finally freeze-dried to recover the product. A precipitation method was exploited to synthesize the AmL-His/HCPT nanoparticles (AmL-His/HCPT NPs), which had an average diameter of 38 nm, using DMSO and water as an antisolvent. A phosphate buffer saline (PBS) solution was used at three pH values (7.5, 5.5 and 4.5) to monitor the release of AL-His/HCTPT NPs in vitro. The authors discovered that the HCPT release speed under physiological conditions is slower than under weakly acid conditions, revealing the pH responsiveness of the system. In addition, AL-His/HCPT NPs are characterized by considerable biocompatibility, efficient cellular uptake (∼twice higher than that of pure HCPT) and positive drug-loading performance (∼15.57 wt%). In vivo antitumor activity studies on a 4T1 tumor-bearing mice model revealed excellent anti-tumor effects (tumor growth inhibition rate: about twice higher than that of pure HCPT) and diminished side effects in the group treated with the NPs.

Wang et al. [88], have exploited microwave irradiation to assist the incorporation of silver nanoparticles (AgNPs) into quaternized alkali lignin (QAL) to enhance the antibacterial activity of the AgNPs via the electrostatic capture. To prepare the QAL, alkali lignin was dissolved in a sodium hydroxide solution and heated at 85–90 °C in the presence of 3-chloro-2-hydroxypropyltrimethylammonium chloride (CHMAC) for 4 h. The positively charged QAL serves as both a stabilizing and reductive carrier, but also endows the system with electrostatic effects toward negatively charged *Escherichia coli* (*E. coli*) and *Staphylococcus aureus* (*S. aureus*). AgNP encapsulation was carried out by reacting a silver nitrate (AgNO_3_) solution and water dispersed QAL under microwave radiation at 320 W for 10 min. The evaluation of the antibacterial activity of the synthesized nanocarrier against Gram-negative *E. coli* and Gram-positive *S. Aureus* revealed 3.72 log_10_ (>99.9%) and 5.29 log_10_ (>99.999%) CFU/mL reductions against *E. coli* and *S. aureus*, respectively, after 5 min. In addition, Ag@QAL also proved environmentally benign as the release of silver was negligible, thanks to the tenacious interaction between Ag^+^/AgNPs and QAL. 

Despite the extensive use of lignin-loaded nanodrug carriers alone, drug-loaded LNPs can also be encapsulated into nanofibers for efficient cervical cancer-cell inhibition, as suggested by Li et al. [87]. In particular, the authors first loaded paclitaxel (model drug) into lignin nanoparticles and, subsequently, encapsulated, using the electrospinning technique, the paclitaxel-LNPs into a composite and biocompatible nanofibrous membrane that was composed of poly(vinyl alcohol)/polyvinyl pyrrolidone for drug-carrier purposes. In vitro experiments revealed that the electrospinning process improved both the drug-release profile and the hydrophilicity of the nanofibrous membranes, leading to strong cell adhesion and proliferation. Moreover, a positive cell inhibition ability (21% cellular survival rate at day 7) was observed at the end of cellular experiments. 

## 4. Outlook: “How Far Is Lignin from Being a Green Carrier for Bioactive Compounds?”

**Figure 10:**
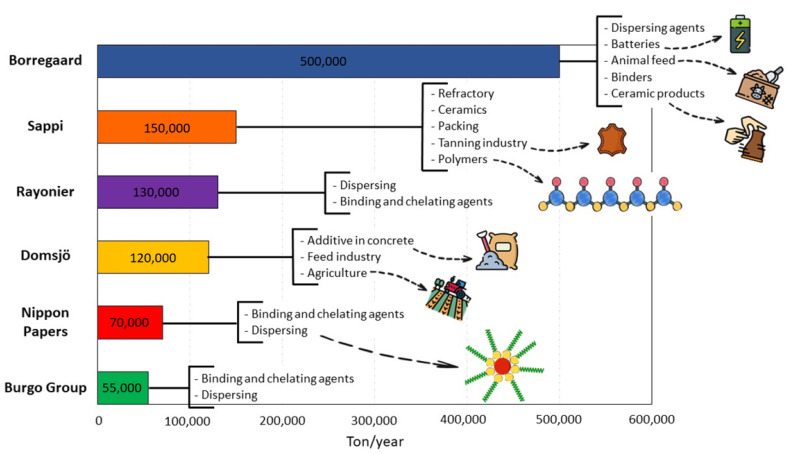
Commercial lignin brands and their applications.

**Figure 11:**
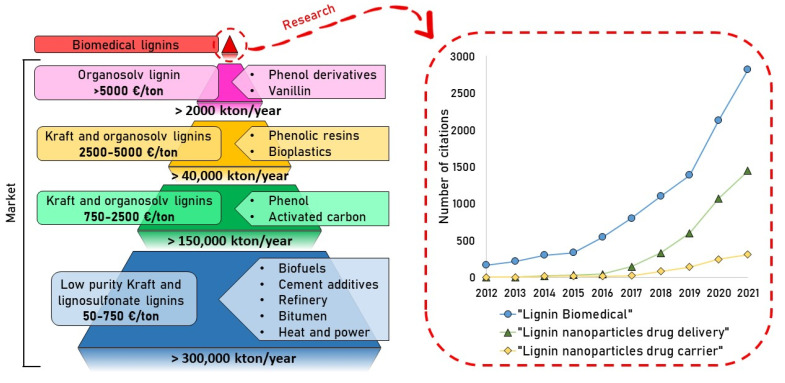
The lignin market in the last ten years (**left**) and the number of citations per year (**right**) related to the use of lignin in the biomedical field (Web of Science^TM^).

The valorization of lignocellulosic biomass currently almost exclusively focuses on the production of pulp, paper and bioethanol from its holocellulose constituent, while the remaining lignin part, which possesses the highest carbon content, is burned and treated as waste. Although no market for profitable businesses using technical lignin yet exists, the future perspectives are outstanding in the short term. Pulping companies (such as LignoBoost^®^ and LignoForce^TM^) produce around 100 million metric tons of Kraft and other technical lignins per year worldwide. However, less than 2% of these lignins are commercialized, mainly as adhesives, surfactants and dispersant formulations [89] (Figure 10). Lignosulfonates are by far the most important lignin derivative, with a production of 1.3 million metric tons per year, whereas just 265,000 metric tons of Kraft lignin are isolated and used for biorefining applications today. Moreover, soda lignin and hydrolysis lignin from cellulosic ethanol production are increasing in their importance and market share, with 75,000 metric tons being produced per year [90]. Moreover, thanks to huge investments (by Stora Enso, the largest producer of Lineo^TM^), Kraft lignin is currently the most important one, with an impact on some emergent economies and even in developed countries [91]. Hence, these lignin sources must be studied in detail in order to provide options for the development of new profitable businesses (Figure 11).

New and exciting applications for lignin have been developed for the biomedical field in recent years and have been discussed in this review (Table 1). It is thus rational to foresee that lignin-based smart materials will continue to see rapid expansion towards new materials and applications in the next few years. However, there are future challenges for lignin applications in the biomedical field, including gaining a better understanding of its structure, isolation and predictable batch-to-batch properties in order to achieve “standardized” building blocks. Furthermore, structure–function relationships for applications in human health are also important. 

There is a plethora of lignin types that are currently being exploited for emerging health purposes, as highlighted in this review, and these arise from different sources and processes, which makes the application of lignin as a standardized smart and green material in biomedical applications even more challenging (Table 3). Moreover, there is no “immediate” (or ready to start) clean source of lignin that can be directly utilized as a biomaterial, although organosolv lignin may be a potential candidate here due to its cleaner extraction process, which uses green solvents.

Demonstrating the scale-up feasibility of standardized lignin for biomedical application is also important to attract industrial activities and applications in this new material domain. As reported by Ragauskas et al. (2014) [34] “one can make everything from lignin except money”. In fact, no patents have been documented for lignin biomedical applications to date.

Nevertheless, the degradability of lignin in the human body is still unclear and future studies exploring this area are also necessary [73]. Furthermore, more studies are required to obtain fundamental knowledge of lignin’s impact on cells, proteins and genes before its widespread application in human health.

Although lignin acts mainly as an inert support in most of the herein reported examples, it is a new material that should be exploited for health purposes, meaning that there is a need for information on its structure-activity relationships, which is currently lacking. 

In particular, lignin modification showed a critical role in the development of new biomedical materials. The main modification strategies lead to lignin hydroxyl groups functionalization by alkylation/dealkylation, esterification, etherification, and phenolation. These conversions could drastically modify lignin properties and the potential application as a drug carrier. Recent works on lignin-based biomaterials usually briefly described the crucial effects of lignin structure on its application. Nevertheless, with a better understanding of its properties, it would also be possible to boost the development of lignin-based biomaterials through the exploitation of its well-known antimicrobial and antioxidant properties.

## 5. Conclusions

The synthesis of lignin nanoparticles is emerging from the recent valorization pathways described for lignocellulosic biomass in the field of biomedical applications. However, the high biodiversity of lignin sources and the lack of standardized procedures for its extraction for biomedical purposes mean that drug-delivery applications are relatively newly documented in the literature. 

With lignin being a relatively new biomaterial, limited data on physico-chemical properties are available to effectively compare other biomaterials. Only thorough studies on the structure/activity relationship and a better mechanisms understanding of carrier-drug interaction, could shed light in the field of alternative biomaterial. In vivo biocompatibility and consequent biodegradability have to be better investigated, including lignin’s impact on cells, proteins, and genes. Moreover the heterogeneity of natural lignin makes their applications troublesome. Last but not least, the mechanism of lignin breakdown by the human body remains one of the open challenges for a safe use of lignin as a biomaterial.

An extensive study of the fundamental nature and properties of lignin is still required, but the application of this “waste” for health purposes may be a topic of wide interest in the near future.

## Figures and Tables

**Table 1:** Case studies of lignin biomedical application.

Carrier Material	Lignin Source	Active Substance	Reference
**Magnetic LNPs**	Alkali Lignin	Resveratrol (RSV)	[14]
**Lignin/chitosan NPs**	Corncob lignin (formic acid pretreatment)	Curcumin (CCM), Docetaxel (DTX)	[77]
**Blank LNPs**	Kraft Lignin	Irinotecan	[78]
**Acetylated and Benzoylated LNPs**	Pine Alkali Lignin	Avermectin (AVM)	[79]
**Sodium lignosulfonate-cetrimonium bromide NPs**	Alkali Lignin	Avermectin (AVM)	[80]
**High and Low molecular weight LNPs**	Kraft Lignin	Cholesteryl chloroformate (CholCl)	[81]
**LNPs, Iron (III)-complexed LNPs and Fe3O4-infused LNPs**	Softwood Kraft lignin	Benzazulene (BZL), Sorafenib (SFN), Capecitabine(CAP)	[82]
**High molecular weight LNPs**	Alkali Lignin	Silver (Ag^+^)	[83]
**LNPs**	Acidolysis lignin from beech wood sawdust	Essential Oils	[84]
**LNPs**	Alkali lignin	Ibuprofen (IBU)	[85]
**Lignin-histidine conjugate NPs**	Aminated Lignin	10-hydroxycamptothecin	[86]
**Quaternized LNPs**	Alkali lignin	Silver nanoparticles (AgNPs)	[87]
**LNPs into poly (vinyl alcohol)/polyvinyl pyrrolidone nanofibrous membrane**	Not available	Paclitaxel	[88]

**Table 2:** Essential-oil loading, drug-loading efficiency (DLE) and drug-loading capacity (DLC) of LNPs.

EO-LNPs Carrier	EO (mg/mL)	Lignin (mg/mL)	DLE (%)	DLC (%)
Cinnamon bark	4.7	15.0	35.5	29.6
Common thyme	9.1	14.0	70.0	60.2
Wild thyme	7.7	14.3	60.7	43.9

**Table 3:** Lignin type: characteristics and applications.

Lignin Type	Source	Application	Characteristics	Solubility	MW (Da)	Aliphatic OH (%)	Phenolic OH (%)	N (%)	S (%)	PDI
Soda	− Annual plants	− Anti-UV, antioxidant, and antimicrobial activities − **Biomedical**	− Sulfur-free − p-hydroxyl units and carboxyl groups − High Ni and silicate content	Alkali	1300–10,400	2.5–3.1	4.4	0.17	0	2.5–3.5
Organosolv	− Softwood − Hardwood − Annual plants	− Anti-UV, antioxidant, and antimicrobial activities − **Biomedical**	− Sulfur-free − High purity − Very hydrophobic	Organic solvents	4100–10,800	3.2–3.5	2.7	0.02	0	1.5–2.5
Lignosulfonate	− Softwood − Hardwood	− Not for biomedical for high sulfur content − **Anti-coagulant** − **Antitumour** − **Antiviral** − **Anti-ulcerogenic**	− Self association and agglomeration in aqueous solution − High S content and MW	Water	12,000–60,000	-	2.0	0.02	3.5–8	6–8
Kraft	− Softwood − Hardwood	− Anti-UV, antioxidants and antimicrobial activities − **Biomedical**	− High phenolic OH content − Low S content	− Alkali − Organic solvents	3700–19,800	9.8–10.1	4.5	0.05	1–3	2.5–3.5

## Data Availability

Not applicable.

## Abbreviations

ACAL	Acetylated Lignin
Ag@QAL	silver-loaded quaternized alkali lignin
AgNPs	silver nanoparticles
AIKL	acetone insoluble fraction of alkali lignin nanoparticles
AIKL-chol	cholesteryl-loaded acetone insoluble fraction of alkali lignin nanoparticles
AL/RSV/Fe_3_O_4_ NPs	resveratrol-loaded and magnetic alkali lignin nanoparticles
AL	alkali lignin
AmL	Aminated lignin
AmL-HIS/HCPT NPs	10-hydroxycamptothecin-loaded aminated lignin-histidine conjugate nanoparticles
ASKL	acetone soluble fraction of alkali lignin nanoparticles
ASKL-chol	cholesteryl-loaded acetone soluble fraction of alkali lignin nanoparticles
AVM	avermectin
AVM@ACAL	avermectin-loaded acetylated Lignin
AVM@BZAL	avermectin-loaded benzoylated Lignin
BBD	Box–Behnken experimental design
BLNPs	Blank Lignin Nanoparticles
BTMAC/FA	Benzyltrimethylammonium chloride/formic acid
BZAL	Benzoylated Lignin
BZL	Benzazulene
BZL-pLNPs	Benzazulene-loaded pure lignin nanoparticles
CAP	Capecitabine
CCM	Curcumin
CCM@Lig/Chi NPs	Curcumin lignin/chitosan nanoparticles
CEL	cellulolytic enzyme lignin
ChCl	Choline chloride
CHMAC	3-chloro-2-hydroxypropyltrimethylammonium chloride
CholCl	cholesteryl chloroformate
CNCs	cellulose nanocrystals
CTAB	cetrimonium bromide
DESs	Deep eutectic solvents
DETA	Diethylenetriamine
DLC	drug loading capacity
DLE	encapsulation efficiency
DLS	Dynamic Light Scattering
DMF	N, N-Dimethylformamide
DMSO	dimethyl sulfoxide
DTX	Docetaxel
DTX@Lig/Chi NPs	Docetaxel lignin/chitosan nanoparticles
EDC·HCl	1-(3-dimethylaminopropyl)-3-ethylcarbodiimide hydrochloride
EMAL	enzymatic mild acidolysis lignin
EO	essential oil
EO-LNPs	essential oil-loaded lignin nanoparticles
ETA	ethanolamine
Fe(OiPr)_3_	iron (III) isopropoxide
Fe_3_O_4_-LNPs	Fe_3_O_4_-infused lignin nanoparticles
Fe-LNPs	Iron (III)-complexed lignin nanoparticles
HBA	hydrogen bond acceptor
HBD	hydrogen bond donor
HCPT	10-hydroxycamptothecin
HIS	histidine
ILs	ionic liquids
LA	lactic acid
LCSN	Lignin Capped Silver Nanoparticles
Lig/Chi NPs	lignin/chitosan nanoparticles
LNPs	lignin nanoparticles
MWL	milled wood lignin
NaDES	Natural deep eutectic solvent
NHS	*N*-hydroxysuccinimide
pLNPs	pure lignin nanoparticles
QAL	quaternized alkali lignin
RSV	Resveratrol
SFN	Sorafenib
SL/CTAB	sodium lignosulfonate cetrimonium bromide system
SL	sodium lignosulfonate
THF	tetrahydrofuran
XNPs	xylan nanoparticles
